# Aqueous Extracts of the Edible *Gracilaria tenuistipitata* are Protective Against H_2_O_2_-Induced DNA Damage, Growth Inhibition, and Cell Cycle Arrest

**DOI:** 10.3390/molecules17067241

**Published:** 2012-06-13

**Authors:** Jing-Iong Yang, Chi-Chen Yeh, Jin-Ching Lee, Szu-Cheng Yi, Hurng-Wern Huang, Chao-Neng Tseng, Hsueh-Wei Chang

**Affiliations:** 1 Department of Seafood Science, National Kaohsiung Marine University, Kaohsiung 811, Taiwan; 2 Department of Biotechnology, Kaohsiung Medical University, Kaohsiung 807, Taiwan; 3 Institute of Biomedical Science, National Sun Yat-Sen University, Kaohsiung 804, Taiwan; 4 Department of Biomedical Science and Environmental Biology, Graduate Institute of Natural Products, Center of Excellence for Environmental Medicine, Kaohsiung Medical University, Kaohsiung 807, Taiwan; 5 Cancer Center, Kaohsiung Medical University Hospital, Kaohsiung Medical University, Kaohsiung 807, Taiwan

**Keywords:** DNA damage, antioxidant, comet assay, red algae, cell cycle arrest

## Abstract

Potential antioxidant properties of an aqueous extract of the edible red seaweed *Gracilaria tenuistipitata* (AEGT) against oxidative DNA damage were evaluated. The AEGT revealed several antioxidant molecules, including phenolics, flavonoids and ascorbic acid. In a cell-free assay, the extract exhibited 1,1-diphenyl-2-picrylhydrazyl (DPPH) radical scavenging activity that significantly reduced H_2_O_2_-induced plasmid DNA breaks in a dose-response manner (*P* < 0.001). The AEGT also suppressed H_2_O_2_-induced oxidative DNA damage in H1299 cells by reducing the percentage of damaged DNA in a dose-response manner (*P* < 0.001) as measured by a modified alkaline comet-nuclear extract (comet-NE) assay. The MTT assay results showed that AEGT confers significant protection against H_2_O_2_-induced cytotoxicity and that AEGT itself is not cytotoxic (*P* < 0.001). Moreover, H_2_O_2_-induced cell cycle G2/M arrest was significantly released when cells were co-treated with different concentrations of AEGT (*P* < 0.001). Taken together, these findings suggest that edible red algae *Gracilaria* water extract can prevent H_2_O_2_-induced oxidative DNA damage and its related cellular responses.

## 1. Introduction

The red algal genus *Gracilaria* is distributed worldwide and is the main source of large scale production of food grade agar and phycocolloids [1]. The global use and the importance of *Gracilaria* are well documented [2]. Currently, Taiwan is one of the few countries that produces *Gracilaria*. Because Taiwan can produce more than 30,000 tons of *Gracilaria* annually, this inexpensive algae is now an important aquaculture species [1]. In Taiwan, *Gracilaria* species have been cultivated since 1961 [3]. The major *Gracilaria* species produced by open sea cultivation are *Gracilariatenuistipitata*, *Gracilaria coforvoides*, *Gracilaria gigas*, *Gracilaria chorda* and *Gracilaria compressa*.

To date, algae represent about 9% of marine biomedical compounds [4]. Moreover, seaweeds are well known sources of bioactive primary and secondary metabolites [5]. Many *Gracilaria* seaweeds, such as *G. gigas* [6], *G. dura* [7] and others [8], contain abundant amino acids, fatty acids, vitamins, minerals, polyphenolic compounds and carbohydrates.

Bioactivities of marine algae of the genus *Gracilaria* have been extensively studied [8]. The many health-promoting properties of genus *Gracilaria* seaweed extracts include the anti-hypercholesterolemic properties of *G.tenuistipitata* [9], the antioxidative properties of *G. tenuistipitata* [10], *G. edulis* [11], *G. salicornia* [12], *G. birdiae* and *G. cornea* [13], the anti-inflammatory properties of *G. verrucosa* [14] and *G. cornea* [15], and the antimicrobial properties of *G. salicornia* [12] and *G. tenuistipitata* [16]. Of these, the antioxidant properties may be helpful for modulating the effects of reactive oxygen species (ROS) generated by cellular metabolism or environmental factors. However, the potential protective effects of *Gracilaria* against ROS are seldom addressed.

The ROS induce formation of 8-oxoguanine, the accumulation of which can induce base pairing mismatch, protein miscoding, DNA mutation and even further genome instability [17]. Excessive free radicals are known to inflict cellular injuries such as gene dysregulation, protein function alteration, lipid oxidation, DNA damage and mutation, and cell growth retardation [18]. Accordingly, reduction of its ROS content and attenuation of its associated effects are important research issues.

This study hypothesized that *Gracilaria* extract has the potential to modulate H_2_O_2_-induced DNA damage and its related cellular responses. The hypothesis was tested by using hydrogen peroxide (H_2_O_2_) as a DNA damaging agent [19] to examine the antioxidative properties of aqueous extracts of *G. tenuistipitata* (AEGT) and its role in recovery from plasmid and cellular DNA damage, cytotoxicity, and cell cycle arrest.

## 2. Results

### 2.1. Polyphenols, Flavonoids, and Ascorbic Acid Contents of AEGT and Its DPPH Radical Scavenging Activity

**Figure 1:**
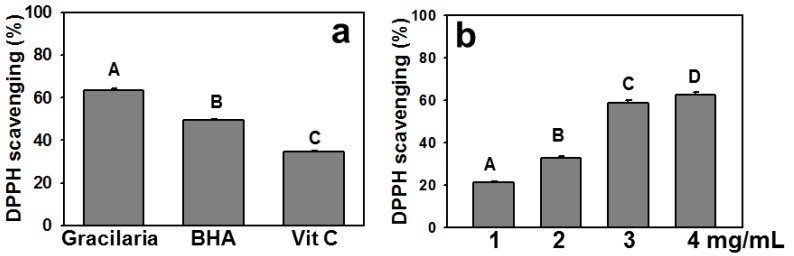
DPPH radical scavenging activity of AEGT. (**a**) Comparison of DPPH radical scavenging in AEGT (4 mg/mL), positive control BHA (10 ppm) and positive control Vit. C (100 ppm). (**b**) DPPH radical scavenging activity of AEGT at different concentrations (1–4 mg/mL). Data are means ± S.D. (n = 3). Levels not connected by same big letter significantly differed. With the exception of A *vs.* D (*P* < 0.05), all comparisons revealed significant differences (*P* < 0.0001).

The amounts of total polyphenol, flavonoid, and ascorbic acid were recorded as 98.94 ± 2.43 μg gallic acid equivalent, 22.59 ± 1.08 μg quercetin equivalent and 1.59 ± 0.18 μg ascorbic acid/mg dry extract, respectively. The DPPH scavenging activity of 4 mg/mL AEGT exceeded 60% (63.37 ± 0.91%), which was significantly higher than those of 10 ppm BHA and 100 ppm ascorbic acid (*P* < 0.0001) (Figure 1a). Moreover, AEGT significantly (*P* < 0.05) increased DPPH scavenging activity in a dose-dependent manner (Figure 1b).

### 2.2. AEGT Modulates H_2_O_2_-Induced Plasmid DNA Strand Breaks

**Figure 2:**
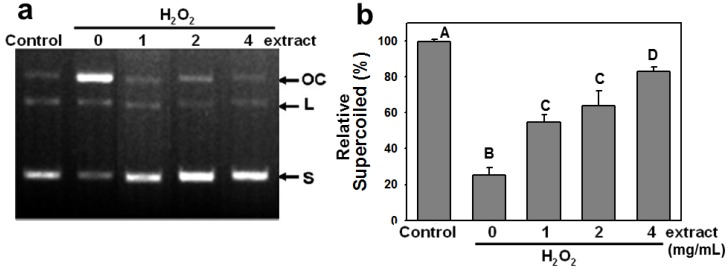
Modulating effect of AEGT on H_2_O_2_-induced plasmid conformational change. (**a**) Gel view of plasmid DNA cleavage assay. The OC, L and S indicate the open circular, linear, and supercoiled plasmid forms, respectively. (**b**) The supercoiled content. Plasmid DNA pBR322 was incubated at 37 °C for 30 min with 0.003% H_2_O_2_ with and without varying AEGT doses (1, 2, and 4 mg/mL). Control was plasmid alone without treatment with H_2_O_2_ or AEGT. Data are means ± S.D. (n = 3). Levels not connected by same big letter significantly differed. Except A *vs.* D (*P* < 0.02), all comparisons among different letters showed significant differences (*P* < 0.005).

This study assessed the protective effect of AEGT against pBR322 plasmid DNA cleavage induced by H_2_O_2_ treatment. In the absence of H_2_O_2_, plasmid DNA appeared mainly in supercoiled form (S) (Figure 2a, control). Adding FeSO_4_ and H_2_O_2_ converted the supercoiled form to the relaxed open-circular (OC) and linear forms (L) (Figure 2a, lane 2). However, 1 to 4 mg/mL AEGT retained the supercoiled form of pBR322 in a dose-dependent manner (Figure 2a, lanes 3–5). The relative percentages of supercoiled plasmid DNA decreased to 25.2 ± 4.3 after H_2_O_2_ treatment and recovered to 54.6 ± 4.1, 63.7 ± 8.3, and 82.9 ± 2.5 in the presence of 1, 2, and 4 mg/mL AEGT, respectively (*P* < 0.02 to 0.001 between H_2_O_2_ alone and 1 to 4 mg/mL AEGT treatment) (Figure 2b). These results indicate that AEGT stabilizes DNA molecules by neutralizing oxidative DNA damage by H_2_O_2_.

### 2.3. AEGT Modulates H_2_O_2_-Induced Cellular DNA Damage

**Figure 3:**
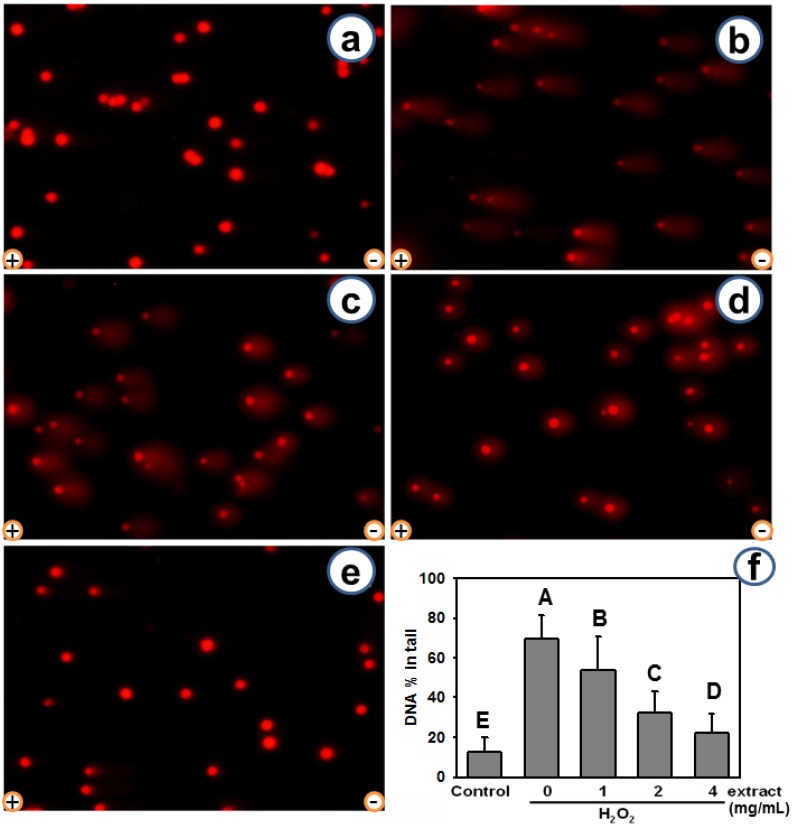
Modulating effect of AEGT on H_2_O_2_-induced cellular DNA strand breaks revealed by comet assay. After 2 h pretreatment with 1, 2, and 4 mg/mL AEGT, H1299 cells were co-incubated with or without 0.003% H_2_O_2_ at 37 °C for 2 h. (**a**–**e**) Demonstration of PI staining results for H1299 cells incubated with or without 0.003% H_2_O_2 _ and AEGT. (**a**) Negative control. (**b**) Positive control (H_2_O_2_ alone). (**c**–**e**) H_2_O_2_ treatment with 1, 2, and 4 mg/mL AEGT, respectively. (**f**) Average % of tail DNA for H1299 cells. Data are means ± S.D. (n = 2; 100 cell counts per experiment with two individual replicates). Levels not connected by same big letter significantly differed (*P* < 0.0001). Electrode polarity is also shown.

Next, the protective effect of AEGT against oxidative DNA damage was verified by comet-NE assay in H1299 cells. Few “tails” were observed in the untreated controls (Figure 3a). While DNA trailing was evident in the presence of H_2_O_2_ (Figure 3b, H_2_O_2_ only), it was reduced by the AEGT co-treatments (Figure 3c–e). The average % in tail values (mean ± S.D.) for NE buffer control, H_2_O_2_alone, and AEGT treatments (1, 2, and 4 mg/mL) were 12.4 ± 7.4, 69.6 ± 11.9, 53.6 ± 17.1, 32.5 ± 10.5, and 22.1 ± 1.0 (n = 100; in duplicate), respectively (Figure 3f, *P* < 0.0001 between H_2_O_2_ only and 1–4 mg/mL AEGT-treated cells). Again, AEGT showed a dose-dependent protective effect against oxidative DNA damage. These results indicate the DNA modulating effect of AEGT in the cellular DNA context.

### 2.4. AEGT Promotes Cell Survival under H_2_O_2_ Treatment

**Figure 4:**
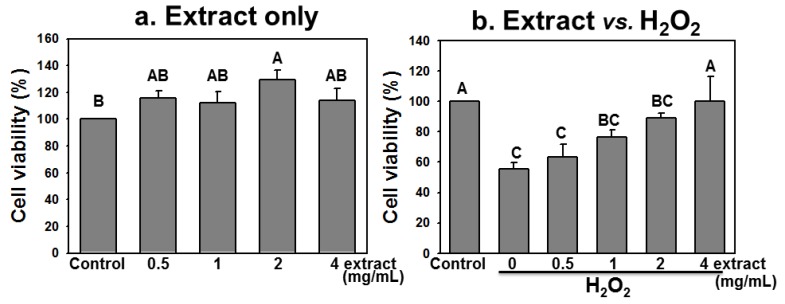
Cell viability was determined by MTT assay. The H1299 cells were treated for 24 h with AEGT (0.5, 1, 2, and 4 mg/mL) (**a**) without H_2_O_2_ and (**b**) with 0.003% H_2_O_2_. Data are means ± S.D. (n = 3). Levels not connected by same big letter significantly differed. Except (**b**) A *vs.* BC (*P* < 0.05), comparisons between other different letters in (**a**) and (**b**) showed significant differences (*P* < 0.005).

We further tested whether the modulating effect of AEGT also promotes H1299 cell survival under H_2_O_2_ treatment. The H1299 cells were treated with or without H_2_O_2_ in the presence of 0.5, 1, 2, and 4 mg/mL of AEGT for 24 h. The cell viability was then determined by MTT assay. Treating H1299 with 0.5, 1, and 4 mg/mL AEGT alone showed no significant adverse effects on cell viability (*P* > 0.05 versus control) (Figure 4a). In the presence of H_2_O_2_–induced oxidative DNA damage, AEGT increased cell viability in a dose-dependent manner. From a baseline cell viability of 55.7 ± 3.8, treatment with 0.5, 1, 2 and 4 mg/mK AEGT significantly increased the cell viability of H_2_O_2_-treated cells to 63.3 ± 8.3, 76.5 ± 4.7, 89.1 ± 3.2 and 100.3 ± 16.0, respectively (*P* < 0.05 to 0.005, Figure 4b). Notably, the viability of H1299 cells treated with both H_2_O_2_ and 4 mg/mL AEGT did not significantly differ from that in control cells (*P* > 0.05, Figure 4b).

### 2.5. AEGT Prevents Cell Cycle Arrest by H_2_O_2_

**Figure 5:**
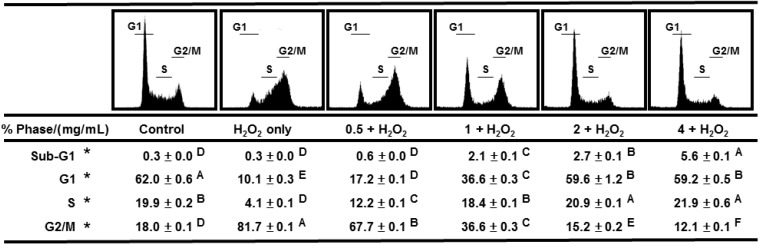
Cell cycle distribution. The H1299 cells were treated with AEGT (0.5, 1, 2, and 4 mg/mL) without and with 0.003% H_2_O_2_ for 24 h. Data are means ± S.D. (n = 3). Levels not connected by same big letter significantly differed (*P* < 0.01 to 0.0001). * The statistical analyses were performed on the same cell cycle phase % between different treatments (control, H_2_O_2_ alone, and AEGT/H_2_O_2_) without comparison between different phases.

After 24 h treatment with H_2_O_2_ in the presence of 0.5, 1, 2, and 4 mg/mL AEGT, the cell cycle distributions of H1299 cells were further analyzed by flow cytometry. The control H1299 displayed a major G1 peak and a minor G2/M peak comprising 62.0% and 18.0% of the cell population, respectively (Figure 5, panel 1). However, the H_2_O_2_-treated H1299 showed a significant increase in the G2/M population to 81.7%, which clearly indicated cell cycle arrest caused by DNA damage (Figure 5, panel 2) (*P* < 0.001). However, this H_2_O_2_-induced G2/M arrest was gradually and significantly diminished by co-incubation with AEGT and was virtually eliminated in the presence of 2-4 mg/mL AEGT (Figure 5, panels 3-6) (*P* < 0.01). This result indicates that AEGT can prevent DNA damage and ensure a normal cell cycle progression.

### 2.6. Discussion

This study investigated the antioxidant activity of AEGT in terms of its biochemical characteristics, DNA damage protection, cell growth, and cell cycle recovery after treatment with H_2_O_2_ alone or with H_2_O_2_ and AEGT. The selected damaging agent in this study was H_2_O_2 _ because it exhibits either direct (oxidation of its target) or indirect (involving peroxiredoxins) signaling [18] and induction of oxidative stress [20,21,22]. The AEGT effectively suppressed ROS-induced DNA strand break in cell-free assay (Figure 2). Sensitive comet-NE assay of cellular DNA damage (Figure 3) and cell cycle profiling (Figure 5) further showed that AEGT increases H1299 cell survival by modulating oxidative stress (Figure 4). In accordance with many other reports of H_2_O_2_ treatment in various cell lines [23,24,25], H_2_O_2_-treated H1299 also showed prominent G2/M arrest, and AEGT conferred effective recovery from H_2_O_2_-induced G2/M arrest although the sub-G1 population in H_2_O_2_/4 mg/mL AEGT is mildly increased (Figure 5). Earlier reports that ROS correlate with immunity [26], anti-hepatoma activity [27], HCV-related activity [28], and Alzheimer’s disease [29] suggest that AEGT has potential applications in testing recovery from ROS-related cellular responses.

Typical phenolic compounds known to exhibit antioxidant activity include phenolic acids and flavonoids [30,31]. In addition to phenolic compounds, tannins [32], ascorbic acid [33], and pigments [34] are other potential antioxidant compounds contained in seaweeds. In the current study, polyphenols, flavonoids, and ascorbic acid were also detected in AEGT. Algal polyphenols are known to confer the major antioxidant activity of seaweed extracts [35]. Analysis of DPPH radical scavenging is largely performed to assess the free radical scavenging effect of specific compounds or extracts and can quickly indicate antioxidant activity [36,37]. The DPPH radical scavenging and phenolic content reportedly have a strong correlation in plant-based foods such as herbs [38] and seaweeds [39]. Moreover, flavonoids [40] and ascorbic acid [36] also exhibit free radical scavenging activities.

Oxidative DNA damage can result from ROS that are generated by cellular metabolism or environmental stress and it is believed to contribute to aging, carcinogenesis and other diseases [41]. For *In vitro* assays, the conversion of the supercoiled form of plasmid DNA into either open-circular or linear form has been used as a standard indicator of DNA damage [42].

The literature shows that boiled *G. tenuistipitata* extract exhibits radical scavenging activities with an IC_50_ of 24.22 mg/mL in DPPH radical scavenging assay [10]. This study shows that AEGT exhibits an even more potent antioxidant activity. A 60% DPPH scavenging activity was observed at only 3 mg/mL. Considering the rapid growth of seaweed and the extensive use of seaweed extracts in the food and cosmetics industries, AEGT represents an attractive multifunctional alternative for such applications. Future studies to identify the bioactive fraction of AEGT would further enhance the economic value of red algal extract. The potential protective role of AEGT to other types of cancer cells warrants further investigation.

Moreover, many algal extracts reportedly exhibit other interesting biological effects, such as the protective effects of enzymatic extracts from microalgae against H_2_O_2_-induced DNA damage [43] and the protective effects of *Phaeodactylum tricornutum* lipid-rich algae extract against proteasome activity [44]. Similarly, we found that AEGT protects against H_2_O_2_-induced plasmid and cellular DNA damage, cytotoxicity, and cell cycle arrest without affecting cell viability as observed in AEGT alone.

## 3. Experimental

### 3.1. Raw Materials

Specimens of *G.tenuistipitata* collected during spring 2009 from a culture farm at Kouhu beach, Yunlin County, Taiwan, were delivered to the laboratory at 0 °C. In the laboratory, the seaweeds were washed with running tap-water to remove epiphytes and encrusting material, immersed twice in distilled water, and then lyophilized. After pulverizing the dried sample and passing it through a 60-mesh sieve, the lyophilized sample was ground to fine powder and stored at −40 °C.

### 3.2. Extraction and Isolation of Seaweed *G. tenuistipitata*

After the addition of 1,000 mL deionized water, the dried samples (50 g) were agitated in a mechanical shaker at room temperature for 24 h. The extract was then filtered with Whatman No. 1 filter paper. The filtrate solution was evaporated to dryness at 40 ± 2 °C in a rotary evaporator (Buchi Laboratoriums-Technik, Buchs, Switzerland) and then lyophilized. The lyophilized extract was stored in a sealed container at −40 °C until use.

### 3.3. Determination of Total Phenolics, Flavonoid, and Ascorbic Acid of AEGT

The total phenolic compounds in AEGT extracts were determined with Folin-Ciocalteu reagent as described by Singleton and Rossi [45] using gallic acid as standard. The total phenolic content was expressed as gallic acid equivalent in μg per mg of dry sample. Ascorbic acid was quantitatively determined using the 2,6-dichloroindophenol-Na dye method as described by Jones and Hurghes [46]. Results were presented on a dry matter basis (μg ascorbic acid per mg of dry sample). Flavonoid content was determined by the colorimetric method described by Woisky and Salatino [47]. Total flavonoid content was calculated in quercetin equivalents based on a calibration curve and expressed as μg quercetin equivalents per mg of dry sample.

### 3.4. Free Radical Scavenging Activity

The ability of AEGT to scavenge 1,1-diphenyl-2-picrylhydrazyl (DPPH) radicals was determined as described earlier [48]. Briefly, a 1 mM methanolic solution of DPPH (1 mL) was mixed with solution of each extract (1, 2, and 4 mg/mL, 3 mL). After vigorous vortexing, the mixture was kept in darkness for 30 min at room temperature. Absorbance was measured at 517 nm, and activity was expressed as percentage of DPPH scavenging compared to control. The percentage of scavenging activity was calculated as [(A_c_ − A_s_)/A_c_] × 100 where A_s_ is the absorbance measured with the extract sample in the assay and A_c_ is the absorbance of control (without extract sample). Butylated hydroxyanisole (BHA) and ascorbic acid (Vit. C) were used as positive controls.

### 3.5. Cell Cultures

Cells were routinely maintained in complete RPMI-1640 (Gibco, Grand Island, NY, USA) supplemented with 10% fetal bovine serum (FBS) (BenchMark, GEMINI, Bio-Products, West Sacramento, CA, USA), 100 U/mL penicillin, 100 μg/mL streptomycin and 0.03% glutamine (Gibco, Invitrogen Ltd., Carlsbad, CA, USA). Cells were kept at 37 °C in a humidified atmosphere containing 5% CO_2_. The H1299 (human lung adenocarcinoma) cell line was used to test cytotoxicity in the lung cancer cell line.

### 3.6. Plasmid DNA Cleavage Assay

Conversion of the supercoiled (S) form of plasmid DNA to the open-circular (OC) and/or further linear (L) forms was analyzed as an indicator of DNA strand breaks [49]. Reaction mixtures (10 μL) containing 150 ng of pBR322 plasmid DNA, 0.1 mM FeSO_4_, 0.05% H_2_O_2_ and AEGT at concentrations of 0, 1, 2, and 4 mg/mL were incubated at 37 °C for 30 min. After stopping the reaction by adding 2 μL of 6 × gel loading dye (0.05% bromophenol blue, 40 mM EDTA and 50% glycerol (v/v), electrophoresis was performed on 0.8% agarose gel in 0.5 × TAE buffer at 50 V for 1–2 h. The DNA in the gel was stained with ethidium bromide (final concentration 0.8 μg/mL) and then visualized and photographed under ultraviolet light. The formula used for calculating the percentage of supercoiled DNA was as follows: S% = (band density of S / band density of (S + L + OC), where the band density was determined by the Gel-pro Analyzer 4.0 (Media Cybernetics, Bethesda, MD, USA). Relative S% = S% of test sample/S% of control.

### 3.7. Comet-NE Assay

The comet-NE is more sensitive than the traditional comet assay [50,51]. The comet-NE assay using nuclear extracts (NEs) prepared from NB4 cells (human acute promyelocytic leukemia) cell line [52,53,54] was performed using a protocol described previously [52,53,54]. Aliquots (100 μL) of H1299 cell suspensions (1 × 10^6^ cells/mL in PBS) were mixed with equal volumes of 1.2% low-melting agarose (in PBS, pH 7.4) and immediately pipetted onto a glass slide precoated with 1% regular agarose (in distilled water). The slides were then immersed in freshly prepared ice-cold cell lysis solution (2.5 M NaCl, 100 mM EDTA, 10 mM Tris pH 10, 1% *N*-laurylsarcosine, 1% Triton X-100 and 10% dimethylsulfoxide or DMSO) incubated at 4 °C for 2 h and then rinsed three times with deionized water. A 20 μL excision mixture containing 0.6 μg NE, 50 mM Hepes-KOH (pH 7.9), 70 mM KCl, 5 mM MgCl_2_, 0.4 mM EDTA, 2 mM ATP, 40 mM phosphocreatine and 2.5 mM creatine phosphokinase was then added to each slide. After applying the cover slips, the slides were incubated at 37 °C for 2 h in a humidified space for NE digestion. The slides were denatured in 0.3 N NaOH, 1 mM EDTA for 20 min and then electrophoresed at 20 V, 300 mA for 25 min. After washing with deionized water, the slides were neutralized in 0.4 M Tris-HCl, pH 7.5 and stained with 40 μL propidium iodide (PI, 50 μg/mL). Under a fluorescence microscope (TE2000-U; Nikon, Tokyo, Japan), the migration of DNA from the nucleus of each cell was measured with the CometScore [55] software program. The % tail DNA parameter [56,57] was calculated as the percentage of DNA in the comet tail (sum of intensities of pixels in the tail). The formula used for calculating the % tail DNA was as follows: Tail % DNA = 100 − Head % DNA, where Head % DNA = (Head Optic Intensity / (Head Optic Intensity + Tail Optic Intensity)) × 100.

### 3.8. Cell Viability Assay

The 3-(4,5-Dimethylthiazol-2-yl)-2,5-diphenyltetrazolium bromide (MTT) assay was performed as described previously [58]. Briefly, fresh medium (100 μL) containing 0.5 mg/mL MTT was added into each well of a 96-well plate containing 5 × 10^3^ cells/well and incubated for 2 h at 37 °C. After removing MTT-containing medium, 100 μL of DMSO was added into each well to dissolve the purple formazan crystal. The plates were then shaken gently for 20 min in darkness and then read at 595 nm on a microtiter plate reader.

### 3.9. Cell Cycle Histogram Obtained by Propidium Iodide Staining in Flow Cytometry

The cell cycle histogram was determined as described previously [59]. Briefly, cells with 5 × 10^5^ cells/100-mm Petri-dish were plated. After recovery, cells were treated for 24 h with 0, 1, 2, and 4 mg/mL AEGT with or without 0.003% H_2_O_2_. After treatment, cells were collected, washed twice with PBS, and fixed in 70% ethanol overnight. The cells were then centrifuged at 700 rpm for 5 min at 4 °C and then resuspended in PBS buffer containing 10 μg/mL PI (Sigma, St Louis, MO, USA) and 10 μg/mL RNase A. After 15 min incubation in darkness at room temperature, the cells were analyzed with a FACScan flow cytometer (Becton-Dickinson, Mansfield, MA, USA) in cell counts of 10,000 with gated setting (forward light scatter versus side light scatter such that only single cells were assayed) [60], and the gated data were analyzed by Cell-Quest and Modfit softwares (Becton-Dickinson, Mansfield, MA, USA).

### 3.10. Statistical Analysis

All data were presented as means ± SEM. Experimental groups were compared by one-way ANOVA with Tukey HSD Post Hoc Test using JMP® 9 software [61]. Levels not connected by the same big letter significantly differed.

## 4. Conclusions

Taken together, the experimental results in this study confirmed the hypothesis that edible red algae *Gracilaria* extract prevents ROS-induced DNA damage and its related cellular responses.
